# Microbiome-based interventions to modulate gut ecology and the immune system

**DOI:** 10.1038/s41385-022-00564-1

**Published:** 2022-09-30

**Authors:** Thomas C. A. Hitch, Lindsay J. Hall, Sarah Kate Walsh, Gabriel E. Leventhal, Emma Slack, Tomas de Wouters, Jens Walter, Thomas Clavel

**Affiliations:** 1 Functional Microbiome Research Group, Institute of Medical Microbiology, University Hospital of RWTH Aachen, Aachen, Germany; 2 Gut Microbes & Health, Quadram Institute Biosciences, Norwich, UK; 3 Intestinal Microbiome, School of Life Sciences, ZIEL—Institute for Food & Health, Technical University of Munich, Freising, Germany; 4 Norwich Medical School, University of East Anglia, Norwich, UK; 5 School of Food and Nutritional Sciences, University College Cork, Cork, Ireland; 6 APC Microbiome Ireland, School of Microbiology and Department of Medicine, University College Cork, Cork, Ireland; 7 PharmaBiome AG, Zürich, Switzerland; 8 Institute of Food, Nutrition and Health, Department of Health Sciences and Technology, ETH Zürich, Zürich, Switzerland

## Abstract

The gut microbiome lies at the intersection between the environment and the host, with the ability to modify host responses to disease-relevant exposures and stimuli. This is evident in how enteric microbes interact with the immune system, e.g., supporting immune maturation in early life, affecting drug efficacy via modulation of immune responses, or influencing development of immune cell populations and their mediators. Many factors modulate gut ecosystem dynamics during daily life and we are just beginning to realise the therapeutic and prophylactic potential of microbiome-based interventions. These approaches vary in application, goal, and mechanisms of action. Some modify the entire community, such as nutritional approaches or faecal microbiota transplantation, while others, such as phage therapy, probiotics, and prebiotics, target specific taxa or strains. In this review, we assessed the experimental evidence for microbiome-based interventions, with a particular focus on their clinical relevance, ecological effects, and modulation of the immune system.

## Introduction

To fully appreciate the stakes and challenges of microbiome-based interventions, it is important to detail a few important features of gut microbiomes, which is done in this introductory section.

### The gut microbiome is a highly complex ecosystem

The human body is colonised by rich communities of microorganisms at various body sites, with the gastrointestinal tract being home to a cell-rich and diverse community. The intestinal microbiome consists of various types of eukaryotic (fungi, helmiths and protozoa)^1^ and prokaryotic (bacteria and archaea)^2^ microbes, as well as viruses^3^. While protozoa and helminths are frequent colonisers of the gut within developing nations, their prevalence is significantly reduced in developed countries, leading to them being understudied^4^. Both groups have generally been viewed as pathogenic in nature within the human gut, however they have been shown to interact with the gut microbiome and influence the enteric immune system^5^, as well as wider allergic inflammation^6^. Bacteria within the gut have been extensively studied and will be the major topic of this review. Due to the complexity and highly dynamic nature of enteric microbial populations, study of their interactions with the host is made difficult.

With the emergence of large population-based studies from different continents, our understanding of the diverse landscape within the human gut continues to expand^7^. While the human gut may represent a total diversity of ~2000 bacterial species^8^ and almost 200,000 phages^3^, an individual’s gut is likely home to a few hundred bacterial species, although sequencing methods can only detect 100–200 within an individual sample^9,10^. The core human gut microbiome, which are species found in the majority of individuals, consists of less than one hundred out of the thousands of bacterial species found in the intestine^11^. Although these dominant species constitute 99% of the mapped reads in sequencing studies of the human microbiome^11^, each of these core species may be represented by different strains with functional differences, further contributing to the complexity of gut microbiomes^12,13^. Currently it remains unclear how strain-level diversity detected by sequencing translates into physiological differences that are of relevance for functional variations of the entire community between individuals. These issues are further confounded due to much of this variation occurring within species and genes of unknown function^14,15^.

The complex, yet stable gut microbial ecosystem in adults contrasts dramatically with the rapid seeding of the sterile intestinal tract of new-borns. This rapid process of colonisation makes early life an important period during which both the ecosystem trajectory and immune responses are shaped.

### The perinatal period is crucial for establishment of microbe-host interactions

The perinatal period is defined as pregnancy and up to one year post birth and is a time of immense physiological programming that influences health. Although previous studies have suggested an in utero (fetal) and placental microbiome during healthy pregnancies, the detected bacterial ‘communities’ are likely the result of contamination, either during DNA extraction (through contamination of reagents and purification columns), tissue collection, and/or during the sample processing and sequencing^16,17^. Those microbes that have been definitely identified in these otherwise sterile sites include those associated with negative birth outcomes, such as *Streptococcus alginate*^18^. Gut microbes do play a key role in foetal development; however, this is via the maternal gut microbiome, with microbial mediators and metabolites entering the circulation and impacting particular developmental and immune pathways in utero (see sections below)^19^. Interestingly, the maternal gut microbiome alters during pregnancy, including reduction in diversity over the course of gestation and increasing levels of *Bifidobacterium* in women and mice which appears to be partly driven by progesterone^20^. The direct ecological interactions between the gut microbiome and the infant host starts immediately post birth, as this is when initial seeding, from a range of environments and sources, occurs.

Vertical transmission of microbes from mother to baby during vaginal child birth provides a source of *Bacteroides* and *Bifidobacterium* (from the maternal gut) that shapes the initial ecosystem and longer term profiles^21^. Caesarean section delivery (CSD) can interrupt this microbial transfer, and these infants are often characterised by a delay in the acquisition of these commensals, and increased levels of hospital acquired bacteria that may also be multi-drug resistant^22^. As gut bacteria are dependent on the host diet, a diet solely comprising maternal milk drives a *Bifidobacterium* dominant profile (between 50–90% of the total community). This is due to the enzymatic machinery encoded by specific strains of *Bifidobacterium* that are able to break down complex human milk sugars (i.e., human milk oligosaccharides [HMOs]), that are not actively metabolised by the infant^23^. In contrast to the gut microbiome of breast-fed infants, formula-fed infants are characterised by higher microbial diversity and elevated levels of potentially pathogenic organisms, such as *Escherichia coli* and *Clostridioides difficile* (formerly *Clostridium*), and reduced levels of beneficial *Bifidobacterium*^24^.

The introduction of solid food, paralleled with reduced intake of human milk, and then cessation of breast-feeding during weaning results in another highly dynamic phase of ecosystem maturation and structuring. The ever-expanding nutritional content and oral intake of environmental matter drives the introduction, transient and then longer-term colonisation of horizontally acquired microbes. Genera such as *Veillonella, Roseburia*, *Bacteroides*, and members of the *Lachnospiraceae* family begin to establish from month 6 onwards, and although *Bifidobacterium* abundance reduces, there is replacement of strains able to utilise HMOs, with those that are able to use complex plant carbohydrates and starches^25^, leading to the replacement of infant-associated taxa (e.g. *Bifidobacterium longum* subsp. *infantis*) with adult-associated species (such as *Bifidobacterium adolescentis*).

Throughout this perinatal period, the infant gut microbiome is highly dynamic, and therefore more susceptible to microbiome perturbations, which is linked to a plethora of acute and chronic (including immune-mediated) conditions. This is why understanding the ecological principles that govern microbiome assembly during this developmental window is important and a prime-time period for beneficially manipulating the gut microbiome to promote appropriate immune development.

### Equilibrium and imbalance within the gut

The diversity of microbial taxa within an individual’s gut microbiome leads to a large range of microbial communities between individuals. It has been suggested that these communities can be grouped into three categories, originally termed ‘enterotypes’, based on the dominance of species within the genera *Ruminococcus*, *Bacteroides*, or *Prevotella*^26,27^. Since the proposition of enterotypes in 2011, there has been great debate as to if they constitute real ‘clusters’ of the microbial communities or are driven by artificial clustering. While this matter has been reviewed in greater depth elsewhere^28^, enterotypes do not appear to be stable as an individual’s microbiome can swap enterotypes during the course of a year^29^.

While the exact taxa of high importance within the gut are still debated, it is generally agreed that a diverse gut microbiome is beneficial. However, this is a simplistic approach to the microbiome as it is not the diversity of the microbiome per se which is beneficial, but the functions those microbes contribute. In part the association between taxonomic diversity and healthy states is because a diverse community will cover a greater number of functional niches. Furthermore, the interactions between commensal species provide additional benefits that no single species provides on its own.

Despite the apparent simplicity of the gut microbiome that establishes postnatally, important cross-feeding interactions already establish and contribute to the health of the host^30^. Bifidobacteria and lactobacilli produce the necessary enzymes to break down the complex milk oligosaccharides into simpler saccharides like lactose or glucose^31^. These simpler sugars are either absorbed by the host, or alternatively further used as an energy source by, for example, fast growing lactic acid bacteria. These produce lactic acid (or lactate in conjugate form), which itself can be metabolised into short-chain fatty acids (SCFA) such as butyrate and propionate^32^. Thus, even in this early phase of dietary homogeneity a trophic food chain of cross-feeding mechanisms between gut bacteria begins to establish^33^.

Disruption of these metabolic interactions between different gut bacteria can result in disease. For example, the production of hydrogen gas by specific types of lactate-utilising bacteria has been postulated to contribute to infant colic^34^. As the host matures and their diet transitions to solid food, the gut microbiome matures and gains in complexity. This introduces a plethora of novel bacteria that each take on different functional roles, termed niches, within a larger metabolic network that converts primary dietary components (mostly complex fibres, see sections below) into resources for growth or end metabolites^35^.

The occupation of these functional niches, as well as their integration within greater metabolic interactions prevent opportunistic pathogens from being able to colonise. This is termed niche exclusion and occurs if a current occupant of the environment already utilises the same substrates as the pathogen, preventing the pathogen accessing the nutrients it requires. This has been shown to occur with *Klebsiella oxytoca* preventing colonisation of *Klebsiella pneumoniae*^36^, and *E. coli* preventing the colonisation of *Salmonella enterica*^37^. In both cases it was the shared utilisation of specific substrates by the resident microbe which prevented colonisation by the invading pathogen. These examples highlight the benefits of a diverse microbiome, as the more diverse the microbiome is, the more functional niches are occupied and the harder it is for pathogens to colonise.

### Biogeography of the gut is important

The gastrointestinal tract itself is not a homogenous ecosystem but instead consists of a diverse range of environments for microbial species to reside. The small intestine is generally viewed as being sparsely colonised with a limited number of microbial species, which increases towards the distal gastrointestinal tract^38,39^. The limited number of studies that have looked at small intestinal communities suggest the presence of a few dominant taxa such as *Streptococcus*, *Escherichia, Gemella*, and *Veillonella* spp.^40,41^. Distally, the colon is home to a more diverse community of microbes that also varies depending on the segment studied^42^.

While it is the luminal content which is generally studied, the mucosal surface is also home to a distinct community of microbes compared to that of the lumen^43^. A major component of the mucus layer is mucin, a glycosylated protein which can be utilised by specialised species^44^. While mucus in the small intestine and cecum/proximal colon is unstructured and easily detachable, the distal colon is covered in a double layer of dense mucus, limiting direct microbiome-host interaction^45^. This difference in proximity of the host and microbiome is immunologically relevant, with the small intestine acting as the major site of immune priming^46,47^. The mucus also acts as a nutrient source used by the microbiome, facilitating a symbiotic relationship between the host and its microbes which is discussed in more detail later within this review. The mucosal-associated microbiome is enriched with members of *Bacteroidaceae*, *Lachnospiraceae*^48^, *Akkermansia muciniphila*^49^, and *Enterococcus gallinarum*^50^. Similar to the lumen, the mucosal microbiome varies greatly across gut locations, sharing <60% of species across its length^51^.

These differences in composition along the gastrointestinal tract are driven by a range of factors, including the availability of substrates^40^, pH of the environment^52^, the flow of the environment^53^, and partial pressure of oxygen^54^. Simple sugars are absorbed quickly from the fast-flowing content within the small intestine, and oligosaccharides are utilised quickly by the dominant taxa present including lactobacilli^55^, while longer-chain dietary fibres are fermented within the slow-flowing content of the colon^56^. Colonic fibre fermentation is conducted by a diverse community of species, each containing a unique repertoire of carbohydrate utilisation enzymes which work in tandem to release substrates, forming trophic chains^57,58^. Most bacterial species within the human gut are strict anaerobes^59^. Small amounts of oxygen diffuse into the gut lumen from the epithelial cells, leading the mucosal microbiome to contain a greater number of aerotolerant species than the lumen^60^. Disruption of the homeostatic release of oxygen by the host, such as during inflammation, leads to a disturbed microbiota, driven by the growth of facultative anaerobes^61^. For readers interested in the other factors that differ along the biogeography of the human gut, we direct them towards reviews that have covered this subject in greater depth^39^.

Current methods for directly sampling the gut are highly invasive, hence most human microbiome studies are limited to the study of faeces. While the faecal microbiome can provide insights into general shifts within the microbiome, it represents only the end-point of a dynamic process occurring along the gastrointestinal tract. Comparison of its profile to that of other gut locations has shown many species which are dominant throughout the gut are not detected within the faeces by sequencing, however may still be present^51^. Ingestible devices are currently under development that facilitate sampling from sites along the gastrointestinal tract, although further development and testing is required before wide-spread application^62^.

In summary, this introduction aimed to provide key information about the diversity and development of the gut microbiome, as understanding the innate complexity of the system is crucial for the interpretation of how microbiome-based interventions can modify microbial communities for the benefit of the host. The various layers of complexity mentioned above (different microbes; strain-level diversity; many unknown ecosystem members; biogeography of the gut) complicate the understanding of interactions between enteric microbes and the host. Nonetheless, progress has been made in all of these aspects of research, facilitating the implementation of microbiome-based applications in health and disease.

## Methods of microbiome-based interventions, their rationales, and effect on gut ecology

**Fig. 1:**
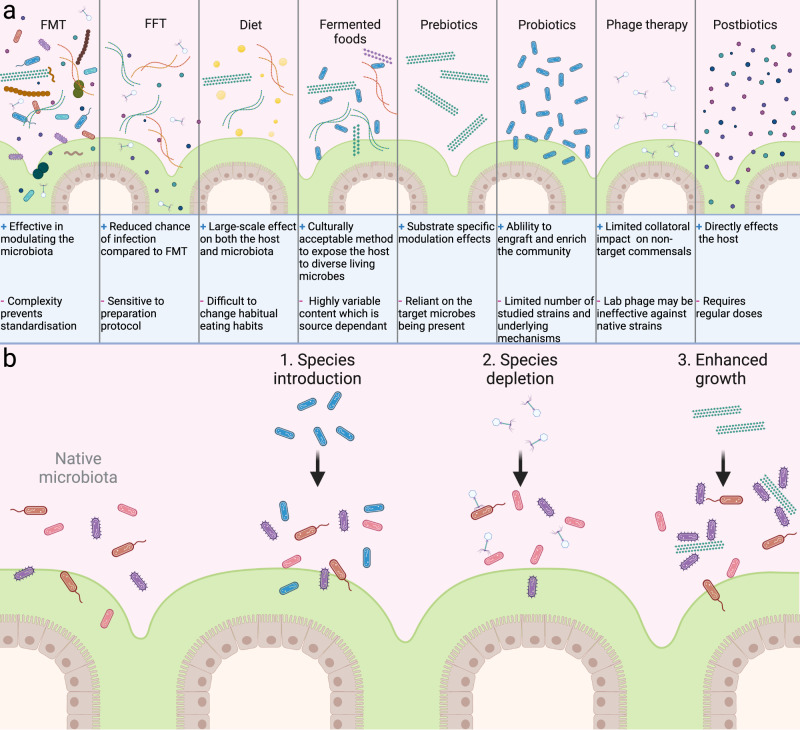
Constituents and mechanisms of microbiome-based interventions. **a** Common constituents of microbiome-based intervention methods are broadly illustrated. **b** Schematic of the three major categories by which interventions influence the gut microbiome. The simplified native microbiome members are coloured shades of red to purple, while introduced species are shades of blue and prebiotic fibres are green. FMT faecal microbiota transplant, FFT faecal filtrate transplant.

The rationales for microbiome-based interventions are diverse, ranging from the prevention of acute infections to the improvement of life-long health (Table 1). Depending on the intervention, the mechanism of action by which the microbiome-based intervention works also vary, from restructuring of the entire community, to supplementation of specific molecules that directly affect the host (Fig. 1a). Based on this, the ecological impact of each intervention can be conceptually defined as belonging to at least one of three categories of ecological impacts: species introduction, species depletion, and enhanced growth (Fig. 1b). For example, phage therapy aims to deplete a species within the ecosystem, hence it can be assigned to the ‘species depletion’ category, while prebiotics are selected based on their specific utilisation by a subset of bacteria, hence they belong to the ‘enhanced growth’ category. Grouping microbiome-based interventions into three categories is a simplistic and reductionist approach as many interventions belong to two or all three categories and any modification to the gut microbiota is likely to have secondary effects.

**Table 1:** Overview of the reasons for microbiome-based interventions.

Rationale	Brief concept	Examples
Prevention of enteric infections	Microbiome-based interventions can help prevent infections via multiple mechanisms. One such mechanism is niche exclusion, which involves commensal species utilising nutrients that the pathogen is specialised into using. Another mechanism is the improvement of barrier functions, which plays a particularly important role in early life. Bacterial production of antagonistic substances such as antimicrobial peptides, or the production of secondary bile acids, have also been shown to be effective in altering susceptibility to infection.	• Reduced epithelial apoptosis during treatment with probiotic strains of *Lacticaseibacillus* spp. (previously *Lactobacillus*) has been proposed as a mechanism for the reduction of necrotising enterocolitis (NEC)^278^. • Colonisation with a closely related strain can lead to competitive exclusion of pathogenic strains. This has been shown for *Klebsiella oxytoca* which prevents colonisation of *K. pneumoniae* colonisation via utilisation of carbohydrates in collaboration with other commensals^36^.
Treatment of infections	While microbiome-therapies can prevent infection, they can also be utilised to treat infections, either by active depletion of the pathogenic species, or by competing for the niche the pathogen occupies.	• Faecal microbiota transplants (FMT) have been utilised globally for treatment of *Clostridioides difficile* infections^64^ with a success rate between 80–94% depending on the method used and cohort^65^. • Phage therapy for the treatment of gastrointestinal infections have been highly effective in animal models^279^, although there have been few clinical trials and those that have been conducted are of limited success^176^.
Gut restoration	Repeated antibiotic use or poor diet over long periods of time can lead to the gut remaining in a perturbed state. Replacement of lost or underrepresented species into the ecosystem can provide the opportunity for the gut ecosystem to recover.	• Meta-analysis of 82 studies confirmed that probiotics can reduce antibiotic-associated ﻿diarrhoea^280^ • FMT has proven effective for improved microbiome recovery from antibiotic treatment^78^
Prevention of chronic diseases	Chronic diseases can develop over a long period of time, with multiple factors influencing their chance to occur. By modulating the contribution by gut microbes, we can reduce the chance of chronic diseases developing.	• Long-term (26 years) intake of dietary fibre has been associated with decreased risk of both ulcerative colitis and Crohn’s diseases^281^. Consistent high fibre intake is essential for this association as other studies that show no association between dietary fibre and inflammatory bowel disease (IBD) incidence utilised a single baseline questionnaire to determine dietary intake rather than regular questionnaires^282^.
Disease treatment	The gut microbiome has been observed to exacerbate certain inflammatory and metabolic conditions. Modification of the microbiome in patients suffering from such conditions may be a route to reduce symptoms and improve patient health.	• The probiotic bacterium, *Hafnia alvei* HA4597, has been shown to reduce feed intake by patients, improving weight loss during dieting^283^. This was linked to the expression of ClpB, a protein similar to α-melanocyte stimulating hormone, which is involved in the regulation of energy balance^284^. • FMT has been studied for inducing remission in patients with ulcerative colitis^285^. Success was achieved in combination with pre-treatment using antibiotics^286^. However, the choice of antibiotics seems important and must be selected with care based on the profiling of antibiotic resistant strains already present within the recipient’s microbiome^287^.
Early modulation of the immune system	The early life gut microbiome coincides with mucosal and systemic immune development—and therefore represents an ideal’window of opportunity’ for targeted modulation of the gut to direct immune programming. This is particularly important in neonates/infants that have ‘perturbed’ microbial ecosystems, such as preterm babies, those born via C-section or who receive antibiotics.	• Human milk oligosaccharides (HMOs) can be deemed prebiotic^181^ due to their bifidogenic effect^288^. *Bifidobacterium* spp. have in turn been shown to be better able to enhance barrier function within the gut when grown on HMOs^289^. Specific probiotic interventions can substantially affect immune responses early in life. For instance, feeding of *B. infantis* EVC001 was linked to upregulation of IFNβ and silencing of intestinal Th2 and Th17 responses^211^.
Improved nutrition	Enteric microbes directly interact with both food components and the host. Some foods have been observed to alter the gut ecosystem in a way to improve the nutritional status of the host. This can be done as part of a holistic diet or via targeted intake of specific nutrients.	• Food specifically designed to modify the gut microbiome for the purpose of enhancing host nutritional status have been developed^290^. The successful application of MDCF-2 to improve growth of undernourished children as well as modification of the gut microbiome confirms the usefulness of such microbiome-targeted approaches^291^. • Variability in glycaemic response to the same diet is linked to a range of microbial functions. Incorporating these variables in a model allowed personally tailored diets to be designed that altered glycaemic response of the individual. The personalised diets had consistent effects on the composition of the gut microbiome, suggesting this may be partly how they influence the host^292^.
Improved vaccine efficacy	The presence of a microbiome has also been shown to influence vaccine efficiency. This can be due to shared sequence similarity between microbes and the sequences targeted by vaccines, priming the immune system and enhancing the immune-stimulatory effect of these vaccines. Another method is that the microbiota can enhance vaccine response via activation of convergent pathways, leading to a greater response than the vaccine would cause alone.	• The application of probiotic bifidobacteria has been shown to enhance the immune response to a neoepitope-based cancer vaccine^293^. This was suggested to be caused by the probiotic leading to an increase in taxa with high sequence similarity to the neoepitope, leading to further immune stimulation. • Activation of TLR5 by the gut microbiota, specifically flagellated bacteria, significantly increases antibody production against influenza vaccination, in mice^294^.
Modulation of drug therapy	The gut microbiome can modify the ability of an individual to respond to medication. This can occur via direct interaction between the microbes and the drugs, e.g., storage or biochemical modification, or by priming the host to be responsive to antibody therapies.	• Bioaccumulation of common drugs has been shown to be a common feature of commensal gut microbes^295^. This may in turn modify the drug dosage received by a patient, preventing clinical improvement^296^. Microbiome modulation to delete species that impact specific drugs may enhance patient response.

For each rationale for microbiome-based intervention we provide details about the concept and published examples.

The impact of each intervention method on the ecology of the gut microbiome are detailed below, along with mechanistic insight into how they can influence the immune system. Each section, based on a single intervention, have been ordered on the complexity of their interactions with both the resident microbes and immune system. As such, FMT is the most complex, consisting of live microbes, remnant dietary components, phage and metabolites. On the other end of the spectrum, postbiotics are purified molecules derived from a microbe, which have a direct effect on either the ecosystem or the host, leading to a desired outcome.

### Faecal microbiota transplant (FMT)

FMT is the administration of stool preparation obtained from a healthy donor to a diseased patient (recipient) with the goal to treat or alleviate the pathology. Such transplants have been done since the 4th century in China^63^, but the last decade has seen an increase in its clinical application, enhancing our understanding. Modern interest in FMT began with the successful application of FMT to treat *Clostridioides difficile* infections^64^. A recent meta-analysis of 45 studies (*n* = 3768 patients) on the efficacy of FMT to treat *C*. *difficile* showed that although highly effective (average success rate between 50.2–96.4%), its success depends on the frequency of transplants, as well as the method of transplantation^65^. The most successful method of transplantation to date is lower gastrointestinal endoscopy (96.4%), with enema being the least effective (50.2%). Repeat administration significantly improved the effectiveness of FMT, independent of the transplantation method.

FMT can lead to community-wide changes in the ecology of the recipient’s microbiome^66^, including the engraftment of species or strains not previously present within the recipient’s microbiome^67^. The effectiveness of FMT to maintain remission in patients with Crohn’s disease is reliant on the engraftment of the donor microbiome^68^. Long-term engraftment of the donor’s microbiome can be difficult to predict as it is dependent on multiple factors including xenobiotics, donor-recipient similarity, and taxonomic profile of the recipient^69^. It has also been suggested that the engraftment of bacteria is not determinative of successful treatment for inflammatory bowel disease (IBD)^70^, which may suggest that, in these cases, the non-living elements (e.g. phage and metabolites) are the beneficial elements (see section on faecal filtrate transplants).

Donor-recipient mapping seems to be a key factor in predicting and increasing the success of FMT. The variability in FMT success has been identified to be partly due to the microbiome of the donor; improved success rate is associated with higher richness as well as the relative abundance of specific taxa, such as *A. muciniphila* and *Ruminococcus* spp.^67^, although such parameters are not universal. It may also be the recipients microbiome which is key, with a depleted recipient microbiome giving the highest chance for engraftment^70,71^. A lower diversity of eukaryotic viruses within the recipients’ faeces has also been suggested to be a marker of FMT success, highlighting the need to screen both donors and recipients prior to transplant^72^. Preliminary findings suggested that the donor’s microbiome profile was the main indicator of success, leading to the concept of ‘super donors’, individuals with a highly diverse microbiome that were most effective for FMT^73^. This concept has been criticised due to lacking consistent experimental support^74^ and ignoring other factors, such as metabolite production by the donor’s microbiome^75^.

To circumvent the issue of relying on donors, autologous FMT (aFMT) has been explored. aFMT refers to patients receiving their own stool, biobanked before undergoing treatment, rather than receiving faeces from separate donors. For instance, when using samples obtained after completing a dietary intervention (Mediterranean or green Mediterranean) and then taken regularly over the course of 6 months afterwards, aFMT improved retention of weight loss and glycaemic control conferred by the diet^76^. aFMT has also been shown to be effective in enhancing recovery of a patient’s microbiome after antibiotic treatment^77^. Further experiments have confirmed that aFMT enhances the recovery of both the luminal and mucosal microbiome after antibiotic treatment^78^.

While the majority of FMT trials have focused on adults, its application during early life has also been explored. Children born via CSD have been identified to have a disturbed microbiome during early-life^79^. This altered microbiome has also been linked to reduced immune priming during the first days of life, which may result in the observed increase in occurrence of immune conditions in CSD children in later life^80^. To avoid this, FMT using faecal samples from the mother has been tested. During an initial trial applying FMT to babies to restore a conventional microbiome, the faeces from 29% of mothers were positive for pathogens, highlighting the need for in depth donor screening^81^. Colonisation of a microbiome deprived environment such as the neonatal gut with pathogens may in turn lead to increased incidence of necrotising enterocolitis (NEC) and other infections. An alternative approach to faecal transfers is the transfer of the vaginal microbiome either via a “swabbing” approach^82^ or oral administration^83^. Potentially due to this variation in approaches, or due to the vagina being a poor source of gut microbes, results have so far been inconsistent.

Ethical concerns have been raised based on the undefined nature of the material during FMT^84^. This has led to increasing interest in the identification of the key species, consortia of species^85^, or molecules that confer the health benefits^86^.

### Faecal filtrate transplant (FFT)

FMT success is generally attributed to the dominant bacterial populations in stool, with a large focus placed on engraftment of donor strains and species^87^. However, the lack of standardisation and use of complex material has been raised as a concern for the wide-spread implementation of FMT. Faecal filtrates aim at removing microbial cells, but retain the viral and small molecules in the donor sample. Faecal filtrate transfer (FFT) was shown to be effective in treating *C*. *difficile* which has been proposed to be due to the transfer of bacteriophages^88^. FFT has also been shown to be more effective than FMT when treating NEC in piglets^89^. Comparison of the effectiveness of faecal sediment *vs*. supernatant for treatment of *C*. *difficile* infection has shown a 27% increase in effectiveness when using the supernatant, confirming that active agents other than the bacterial component are important^90^. However, FFT has been tested in few studies that have relied on a small number of patients. Systematic comparisons between FMT and FFT are required using optimised protocols for each approach (application route for FMT and method of filtration for FFT) before solid conclusions can be made. While FFT has been shown to significantly alter the composition of viruses within the gut^91^, the impact on the entire ecosystem is less understood.

### Diet

There is a large overlap between the chronic diseases linked to diet and those that have been linked to the gut microbiome^92^. These pathologies, such as allergies, obesity (plus metabolic comorbidities), autoimmune, neurological, and oncological disorders, are complex diseases that are to some degree immune mediated with inflammation as a common determinant^93^. Decreased microbial diversity in the gut microbiome is observed both in chronic diseases but also population-wide in Western societies, in which the rates of these diseases are higher^94^. Western diets (WD) are high in processed foods, animal products, and refined grains, and low in whole plant foods such as vegetables, fruits, nuts, and legumes^92^. The impact of a lard-based WD on mice has been shown to be reliant on the presence of a microbiota^95^, as germ-free mice are resistant to obesity from this diet^96^. This interaction between WDs and the microbiota has been confirmed by microbiota transfer experiments into germ-free mice^96^. These showed that microbiota of obese mice significantly increased body-fat gained compared to transfer of a lean mouses microbiota^97^.

Different food components and dietary patterns shape the gut microbiome with associated immune modulating effects. Whole plant foods (fruits, vegetables, wholegrains, legumes, and nuts) are the main source of naturally occurring dietary fibres (see section on prebiotics)^92^. Fermentable dietary fibers are converted into metabolites such as SCFAs which are important mediators for the interaction between the gut microbiome and immune system^98^ and affect the balance between pro- and anti-inflammatory mechanisms. For example, the SCFA butyrate increased the generation of extrathymic T_reg_ cells in mice, additionally, periphery de novo T_reg_ cell generation can be stimulated by the SCFA propionate^99^. Fibre-rich diets also promote a mature intestinal mucus layer and barrier, decreasing pathogen infection and risk of colitis^100^. In contrast, fibre deficient diets are low in available nutrients for the gut microbiome and can promote microbial degradation of mucus, which contributes to the erosion of the colonic mucus barrier, increasing pathogen susceptibility^100^.

The refined food components of processed foods, which dominate WDs, are easily fermented in the small intestine to promote bacterial overgrowth and an undesirable microbial composition and metabolic profile that may negtively influence immune functions^101^ while not being available to colonic microbes^102^. Additionally, the high fat content of WDs may promote the growth of the pathobiont *Bilophilia wadsworthia* which has been associated with inflammation, intestinal barrier dysfunction, glucose dysmetabolism and hepatic steatosis in mice^103^. Emulsifiers present in processed foods such as carboxymethylcellulose have been shown to alter gut microbiota and enhance chronic intestinal inflammation in mice by inducing the expression of gene clusters mediating Crohn’s-disease- associated adherent-invasive *E.coli*^104^. The combined effects WDs negatively influence the composition of the gut microbiome and may weaken mucosal barriers to promote an inflammatory response.

Fruits and vegetables contain a variety of fibres with varying physicochemical properties. Human intervention studies have noted an inhibitory role of fruit and vegetables intake against the growth of pathogenic clostridia (*Clostridium histolyticum*/*perfringens*)^105^ among other positive effects on the microbiome^106^. Whole grains contain a diverse array of hemicellulose fibres, such as xylans and β-glucans, in addition to cellulose, resistant starches, and oligosaccharides^92^. Increasing consumption of wholegrains (whole-grain-barely and/or brown rice) to 60 g/day has been shown to increase microbial diversity and reduce both, plasma interleukin-6 (IL-6) ratio and peak postprandial glucose^107^. The response to increased whole-grain consumption may be highly individualised. Improvements in glucose metabolism in response to consuming a high fibre barley-kernel bread (over 3-days) was associated with an increase in *Prevotella*^27^ in responders (who had a higher ratio of *Prevotella/Bacteroides*)^27,108^.

Dietary patterns such as the Mediterranean diet (MD) combine many universally accepted food-based dietary guidelines and are recommended for their health promoting properties^76^. The MD is characterised by a high intake of plant-based foods, moderate intakes of olive oil, fish and poultry, and low intakes of dairy products, and red meat^109^. This dietary pattern is inversely associated with a reduced risk of cancer and cardiovascular disease^109^. A meta-analysis of 16S rRNA amplicon data (1931 human faecal samples) noted that the gut microbiome associated with the MD was enriched in bacteria with potential anti- Vs. pro-inflammatory properties (e.g. *Akkermansia* Vs. *Fusobacterium*)^110^. Additionally, high level of adherence to a MD was associated with an increase in faecal SCFAs whilst low levels of adherence were associated with higher urinary trimethylamine oxide levels (linked to atherosclerosis and cardiovascular disease)^111^. Due to individual variability and low adherence to prescribed diets, altering their impact, strategies are needed to maintain the impact of diets while being tailored to increase individual adherence^112^.

Ketogenic diets (KDs), restrict carbohydrate intake and provide high levels of fat and adequate protein. KDs have been associated with a reduced incidence of seizures in children with therapy resistant epilepsy^113^ the effects of which may be mediated by the gut microbiome (via bacterial cross-feeding and increases in *Akkermansia* and *Parabacteroides)* through their modulation of hippocampal GABA/glutamate ratios^114^. The restriction of carbohydrate intake, has been shown to be beneficial to patients with non-alcoholic fatty liver disease (NAFLD)^115^. Over 14 days on this diet, community-wide shifts caused significant decrease in SCFA production, but increase in folate production which was associated with decreased liver fat. When compared to restriction of fat intake, patients undergoing carbohydrate restriction for 24 weeks lost significantly more weight^116^. However, compositional shifts observed on KDs may have a less positive influence on gut and overall health, noted by decreases in health promoting (and fibre fermenting) bacteria such as bifidobacteria and *Eubacterium rectale* and their metabolites^113^. Though evidence highlights short-term beneficial effects of KDs in certain population groups, its restriction of fermentable fibre and high fat content may detrimentally impact the gut microbiome and immune response in the long term.

Personalised nutrition focuses on the use of person-specific factors (genetic, phenotypic, medical, nutritional or other relevant information) to develop nutritional recommendations tailored for each individual with the goal to preserve, or increase health^117^. Given the pronounced inter-individual variation of the gut microbiome^118,119^, the microbiome could constitute a key determinant of personalised nutrition. By identifying key microbiome features that predict the response to particular food components personalised nutrition can inform the design of a diet offering favourable health outcomes^120^.

For example, there is emerging evidence that an individual’s microbial composition may influence their ability to lose weight when following specific diets. Stratifying individual’s according to their microbial community may help predict their responses to certain diets^121^. Individuals with a gut microbiome dominated by *Prevotella*^27^ may achieve optimal weight loss by adhering to a high fibre diet^122,123^ which is not observed among individuals with a gut microbiome dominated by *Bacteroides*^121^. The value of a personalized use of diet informed through microbiome information is also supported by the *Prevotella*-dependent^27^ effects of barley-kernel bread on glucose discussed above^108^. However, increasing bifidobacteria in individuals with a gut microbiome dominated by *Bacteroides* has been shown to improve metabolic parameters which could be used as a weight loss strategy^121^. Although the concept of personalised nutritional advice incorporating microbiome analysis is promising, this area of research is still in its early stages with noteworthy limitations including cost and a lack of causal data from clinical trials underpinning recommendations.

### Fermented food

The International Scientific Association for Probiotics and Prebiotics (ISAPP) has defined fermented foods and beverages as “*foods made through desirable microbial growth and enzymatic conversions of food components*”^124^. Fermented foods in which live organisms are present include yoghurt, sour cream, kefir, most cheeses, sauerkraut, kimchi, miso, natto, kombucha, some beers, and non-heat-treated (raw) fermented sausages (e.g. salami). If served uncooked, fermented foods often contain a high number of live microbes and have a long history of safe consumption^124^.

Lactic acid bacteria (LAB) are essential in the production of fermented foods and large quantities of live LAB may be consumed via fermented foods e.g., yoghurt and cheeses. A large-scale genome-wide analysis (analysing 9445 metagenomes from human faecal samples) noted that the occurrence of LAB species was typically low and associated with age, lifestyle, and geographical location^125^. However, it was noted that similar LAB strains occur in both food and gut microbiomes indicating that fermented foods may be the direct source of LAB found in the microbiome. It is important to consider that although live microbes from fermented foods reach the gut microbiome alive^126^, they are not adapted to the gut and thus unlikely to persist and have a major effect on the overall microbial community^127^. Due to this, it is likely that the metabolites produced by these species, which are found in high concentrations within the fermented foods, are the active agents which confer any beneficial effect^128^.

Although the microbes present in fermented foods are unlikely to exert a major impact on the gut microbiome structure, evidence is emerging that they can influence the host and its immune system. In a 17-week randomised prospective study (*n* = 18) during which participants increased their baseline average intake of fermented foods from 0.4 ± 0.6 to 6.3 ± 2.9 servings per day, it was found that a diet high in fermented foods steadily increased gut microbiome diversity and decreased inflammatory markers in the blood^129^. Yoghurt consumption has been associated with a higher relative abundance of species used as yoghurt starters (*Streptococcus thermophilus* and *Bifidobacterium animalis* subsp. *lactis*) in participants who also showed improved metabolic health characterised by reduced visceral fat^130^. Yoghurt contains concentrated milk fermentation metabolites such as branched chain hydroxy acids (BCHA)^131^. Recent studies in mice have noted that feeding the equivalent of 2 servings per day of yoghurt prevented insulin resistance and hepatic steatosis in diet-induced obesity. This was partly driven by changes in gut microbiome composition and maintaining BCHA levels which are typically reduced in diet-induced obesity^131^.

Although there is limited and conflicting evidence that fermented foods contribute to the repopulation and densification of the gut microbiome, they are culturally acceptable and safe vehicles by which live microorganisms can temporarily reside and pass through the human alimentary canal, influencing the immune system. Such foods they may confer health benefits attributed to their low pH and unique preparation methods which expose individuals to microbial species not otherwise present in the food chain.

### Prebiotics

A prebiotic is currently defined as a substrate that is selectively utilised by host microorganisms conferring a health benefit^132^. Although current definitions of prebiotics have been criticised for not allowing a clear distinction between dietary fibres that are prebiotic and those that are not^133–135^, non-digestible carbohydrates (NDCs) can be used to modulate both composition and function of the gut microbiome.

The administration of fibre supplements, no matter if they are considered traditional prebiotics or not, result in specific changes in the gut microbiome^136,137^. These changes are fibre-type specific, meaning, different fibres enrich for different bacterial types. Several studies have established dose-response relationships between the administration of NDCs and compositional shifts^138,139^. At higher doses (10–30 g), the magnitude of the effects of NDCs can be significant, enriching some species by several thousand percent to reach relative proportions of >20% of the gut microbiome^136,138,139^. Using a dose-response trial with three type-IV resistant starches, crystalline and phosphate cross-linked starch structures were shown to induce divergent and highly specific effects on microbiome composition that are linked to directed shifts in the output of either propionate or butyrate^139^, These findings support the concept of precise gut microbiome composition modification through discrete fibre chemical structures^139^. However, these responses are for the most part individualised and reversible when the host is no longer receiving the substrate^104^.

Ecology can be used to explain the effect of dietary fibres and prebiotics. Fibres provide substrates (resources) that select for microbes that are the most adapted to utilising these fibres under the competitive conditions present. The colon consists of a complex ecosystem of microbes in which primary degraders, secondary degraders, and cross-feeders are capable of growing on nondigestible fermentable carbohydrates (NDFCs) and utilising its by-products^140^. Varying microbial species are required to degrade specific fibre types and certain bacteria are highly specialised and significantly contribute to fibre degradation, often referred to as keystone species^104^. Intervention trials with resistant starch, a microbiome accessible NDFC^136,141^, noted significant increases in *Ruminococcus bromii* and *E. rectale* with supplementation. *R. bromii* utilisation of NDFCs can benefit other microbial species by releasing sugars and acetate and can therefore be considered a keystone species^142^. Interestingly, the more complex fibre structures become, the more specific the response. For example, while the effects of high molecular arabinoxylan is highly specific to the species *B. longum* and *Prevotella copri*^27,143^, yet arabinoxylan oligosaccharides seem to have lower specificity and promote multiple species of *Bifidobacterium* and *Prevotella*^27^, as well as several additional genera (e.g., *Eubacterium* and *Roseburia*^144^). Specificity is not only determined by the ability of bacteria to utilise the fibre but also by selective colonisation (attachment) to the substrates^145^.

In addition to impacting the composition of the microbiome, fibre supplements can influence the production of microbial-derived metabolites that influence immune responses (see section on diet). SCFA production is dependent on the fibre type and species present in the host microbiome^104^, providing a framework for directing SCFA production by utilising different fibres^139^ (for a recent review see Vinelli et al.^146^).

Despite the many potential positive effects of fibre supplementation, rodent studies have indicated that high-dose supplementation with certain soluble fibres (such as inulin and pectin) can induce cholestasis, hepatic inflammation and icteric hepatocellular carcinoma (HCC) in mice^147^. Singh and colleagues suggested that the production of excessive amounts of butyrate (beyond levels tolerable to the host) in combination with changes to the microbiota caused by a high fat diet, and inflammation may accelerate tumour formation. In addition, fermentable fibers have been shown to exacerbate severity of DSS-induced colitis in mice^148^. These findings show potential detrimental impact when using certain soluble fibers as supplements in high doses.

The capacity to identify the mechanisms by which specific fibre types regulate host microbial populations and metabolism may offer the possibility for targeted therapeutic dietary fibre interventions to regulate disease states associated with altered metabolism and inflammation^104^. However, well controlled human clinical trials that establish the effects of fibre supplements or prebiotics on the gut microbiome and host health parameters are scarce and effects are highly inconsistent^149^.

### Probiotics

Probiotics are live microorganisms, and thus directly add to the diversity of the native gut ecosystem. The most recent definition of a probiotic is “*live microorganisms that, when administered in adequate amounts, confer a health benefit on the host*”^150^. This definition is purposely broad and inclusive, but the difficulties lie in the demonstration of a health benefit. Probiotics come in a range of complexities, from single strains^151^ to complex microbial assemblages of multiple strains from different species^152^ that are either administered in foods or as supplements.

Traditionally, probiotics have consisted of single strains which may or may not have been isolated from the environment they are applied to. One of the most studied probiotic strains is *Lacticaseibacillus rhamnosus* GG (LGG)^153^. LGG has been used to treat *C. difficile* colitis in humans^154^ and has been shown to restore leptin responsiveness in mice fed a high-fat diet, suggesting it may improve weight loss^155^. When given to mice fed a high-fat diet, LGG was observed to decrease the proportion of Pseudomonadota (formerly Proteobacteria), without impacting overall microbiome diversity. Until recently, probiotics have been limited to a few known taxa (mainly lactobacilli and bifidobacteria), for which multiple strains have been identified and characterised.

Increased activity in cultivating previously unknown taxa from the human gut has provided access to a wider array of strains that may be used as probiotics^2,156–158^. Advances in sequencing technology have also facilitated greater insight into the association of these species and strains with health conditions^159,160^. By utilising the results from sequencing to identify which strains may be beneficial for specific conditions, we can generate probiotics which were not possible before. Probiotics selected via this format have been termed ‘next generation probiotics’ (NGPs)^161^. Due to these strains being selected to treat or prevent specific diseases or conditions, they are likely to fall under the broader category of ‘live biotherapeutic products’ (LBPs) (discussed in Box 1), which would impose a more stringent regulatory framework than those required to be deemed safe as a food supplement and given ‘generally considered as safe’ (GRAS) status. One of the most studied NGPs is *A. muciniphila*, of which one strain (Muc) has been approved, in pasturised form, as a food supplement and categorised as a novel food by the European Food Safety Authority (EFSA)^162^. Ingestion of either live or pasteurized *A*. *muciniphila* have been shown, both in human^163^ and mouse trials^164^, not to cause community wide changes in the microbiome while improving host metabolic parameters including insulin sensitivity and plasma cholesterol levels^163^. In addition to *A*. *muciniphilia*, strains of *Faecalibacterium prausnitzii*^165^ and *Bacteroides fragilis*^166^ have also been studied as potential NGPs, although human trials have not yet been reported. So far, human studies failed to show a significant effect of probiotics on the gut microbiota^167^. This may be in part due to many of these NGP species being strict anaerobes, meaning the higher partial pressure of oxygen within the small intestine could reduce their viability within the distal gut. Engraftment of the probiotic strain into the already present and complex ecosystem is not guaranteed. However, if autochthonous strains are used, permanent engraftment has been reported^168^.

Multi-strain consortia can apply a holistic approach towards probiotics rather than the traditional single strain approach. Including multiple bacterial strains into a single probiotic can have benefits that leverage different approaches. The likelihood of achieving a specific functional target, for example, butyrate production, could be improved by including a number of different strains that have different requirements or interactions with a resident microbiome. The presence of trophic chains within the gut microbiome is one such opportunity, whereby interactions can be manipulated to increase the production of a wanted end-product, such as butyrate. Lactate is produced by many lactobacilli and bifidobacteria as an end-product of carbohydrate fermentation, however, it can then be utilised by butyrate producing species. Supplementation of faecal samples with a multi-strain lactate-producing consortia was observed to cause increased butyrate concentrations after a week^169^. While multi-strain consortia offer a unique opportunity to utilise our knowledge of the microbiome, producing multi-strain probiotics at scale poses technological challenges (see Box 1).

Box 1 Biotechnological trials and tribulations of microbiome therapies

- Traditional probiotics, i.e. lactate producing bacteria, which have been ‘generally considered as safe’ (GRAS) by the US Food and Drug Administration (FDA) and European Food Safety Agency (EFSA), have been broadly used as food supplements. The promise of novel intestinal microorganisms without GRAS status to treat disease has prompted the FDA to define a new regulatory category called ‘live biotherapeutic products’ (LBPs) that fall into the broad definition of probiotics, but are used to treat health conditions. This new category of LBPs are regulated as a drug and are hence subject to the stringent regulations in terms of safety, clinical efficacy, and current good manufacturing practices^297,298^.
- The production of novel single strain probiotics that are classified as LBPs can directly benefit from the experience of producing probiotics at scale, though many strict anaerobic gut bacteria are more fastidious than lactobacilli or bifidobacteria. This requires extra investment to optimise growth conditions during production—even more so for the intestinal microbes that depend on close interactions with other microorganisms for optimal growth.
- The production of multi-strain consortia, however, poses additional biotechnological challenges. Optimal growth conditions will vary between strains, complicating the overall process. When produced as individual mono-cultures that are subsequently mixed in a final product, production complexity scales linearly with the number of strains.
- Alternatively, multi-strain consortia can be directly produced as cocultures. While this addresses the complexity problem of scaling, determining growth conditions that apply to all strains simultaneously is far from trivial. Furthermore, because each strain will typically have different growth rates, guaranteeing reproducibility and consistency of the final product is hard. These challenges can be overcome by incorporating ecological interactions into consortium design^299^.
- The challenge of multi-strain consortium LBPs expands beyond the production process into clinical application, where consistent activity is a requirement for regulators. The control over pharmacokinetics and pharmacodynamics, i.e. growth and exertion of the desired activity of this new modality is currently the biggest challenge that remains to be shown in humans and will be the make or break of the efforts to mimic FMT in a standardised fashion using defined bacterial consortia^300^.

### Phage therapy

Bacteriophages (or phage) are viruses that target bacteria. Most have a narrow host range, meaning they infect closely related strains within a species or related species, limiting their ability to infect other species and reducing collateral damage to the resident microbiome^170^. One example is the development of phage cocktails to treat *C. difficile* infections, which have, in fermenter experiments, been shown to eliminate *C. difficile*, while also avoiding major impact to the commensal groups (bifidobacteria, enterococci, enterobacteria, and lactobacilli)^171^. Phage isolated against disease-associated bacteria, such as Adherent invasive *Escherichia coli* (AIEC), have also been applied to reduce the severity of inflammation in DSS treated mice^172^. Another species targeted to reduce inflammation is *Klebsiella pneumoniae*. A consortium of five phage that target *K*. *pneumoniae* has been developed and shown to reduce the severity of DSS induced inflammation, and an initial human study has shown this consortium is safe for human use, and viable^173^.

However, the interdependency between gut microbes due to cross-species interactions mediated by metabolite production/utilisation can nonetheless lead to collateral effects. Phage treatment against *E. coli* and *Clostridium sporogenes* has been observed to have knock-on effects on non-target species, reducing the abundance of *B. fragilis*, while significantly enhancing the abundance of *Phocaeicola vulgatus*^174^. This in turn modified the metabolome of the gut, which can affect the bioavailability of molecules to the host^175^. While this was observed in mice colonised with a minimal microbial consortium, the impact to a complex community is likely lesser.

The specific nature of phage is also proposed to be a limitation to the application of phage therapy to treat gastrointestinal infections as the phage maintained in culture in laboratories may no-longer be able to replicate in the native pathogenic strains^176^. Phage resistance has also been observed to occur within gut bacterial species, leading to a coexistence between phage and their host, rather than depletion of the latter^177^. Due to their narrow host range and potential coexistence, the ability of phages to modulate the gut microbiome is thought to be limited^173^.

The abundance of phage within the gut has also been linked to increased inflammation caused by IFN-γ activation through a TLR9-dependent pathway^178^. Stimulation of ﻿IFN-γ production from dendritic cells incubated with phage DNA, but not empty phage capsids, confirms that phage DNA is immunostimulatory. The immunogenicity of phage proteins is likely specific to each phage due to the diversity of phage that exist^3^. For example, the T4 phage contains multiple capsid proteins that stimulate an immune response in humans^179^ although safety testing of T4 has confirmed it causes no adverse effect after oral treatment^180^.

While thousands of phage have been identified to exist within the human gut, few have been isolated directly from this environment and fewer still have had their impact on the community studied^3^. Further research is required in this area to understand the direct and indirect impact of phage therapy on both the microbial community and the host.

### Postbiotics

Postbiotics are functionally bioactive molecules produced by microbes, formally defined as a *“preparation of inanimate microorganisms and/or their components that confers a health benefit on the host”*^181^. It is key within this definition that while intact microbial cells may be deemed a postbiotic, the preparation is not alive or viable. This prevents the chance for colonisation and means health benefits conferred by postbiotics are reliant on regular intake to maintain the presence of the bioactive molecules.

An example of an intact cellular postbiotic is the use of pasteurised *A. muciniphila* cells. A trial comparing pasteurised *A. muciniphila* cells to a living culture showed that the pasteurised cells, but not the living cells, improved insulin sensitivity and reduced plasma cholesterol levels^163^. The active molecule responsible for the beneficial effects of pasteurised  *A*. *muciniphila* cells has been identified to be a membrane protein, Amuc_1100^182^.

p40 is another protein which has been identified to be the element conferring the beneficial effects of the common probiotic, LGGs, as it secretes p40 into the environment^183^. While this protein is discussed in greater detail in the next section, in brief it has been shown to improve barrier function^184^. It is possible that this protein influences the ecology of the gut, as administration of LGG significantly increased the richness and evenness of the microbiome when administered during the first week of life^185^.

Direct supplementation of SCFAs as postbiotics for improving mucosal development has been investigated since the 1950’s^186^. As important microbial metabolic molecules, it is not surprising that supplementation of SCFAs can alter the gut microbiome. Butyrate supplementation (300 mg/Kg daily) increased the relative abundance of multiple genera in mice, including *Bacteroides* and *Rikenella*^187^. In humans, 4 g daily butyrate supplementation for a month across 30 individuals was observed to cause a community wide shift, led by *Lachnospiraceae*, including *Dorea formicigenerans*^188^. Similarly, propionate supplementation (1% within drinking water) reduced the impact of high-fat diet in mice, recovering the microbiome richness to control levels and promoting the occurrence of *Bacteroides* and reducing that of *Lactobacillus*^189^.

Secondary bile acids (SBAs) represent another main category of microbial molecules proposed for use as postbiotics, created by modification of primary bile acids released by the host into the gut lumen. The gut microbiome therefore acts as a key modulator of the bile acid pool, which directly regulates certain intestinal immune cell populations^190^. Ursodeoxycholic acid (UDCA) is a SBA produced from chenodeoxycholic acid (CDCA) by a two-step reaction involving 7α- and 7β-hydroxysteroid dehydrogenases (HSDHs)^191^. It contributes a minor fraction of the human bile pool, but is a major component of black bear bile^192^. Traditionally, black bear bile, also called “*Yutan*”, has been used to treat hepatobiliary disorders^193^, but UDCA has been confirmed as the bioactive molecule conferring the beneficial effects. Based on this, UDCA has been widely used as a postbiotic to treat different disorders. UDCA has also been suggested to reduce *C. difficile* associated inflammation via modulation of the host bile pool and via inhibition of *C*. *difficile* spore germination^194,195^. However, unlike antibiotics, UDCA intake caused minor changes to the gut microbiome of human males but not females, even after three years of daily intake^196^. This is in contrast to its impact on the bile acid pool, which after two years of daily treatment with UDCA, a study of 55 patients observed the dominance of cholic acid (CA) in the bile acid pool was replaced with UDCA^197^.

Tauroursodeoxycholic acid (TUDCA) is the conjugated form of UDCA, produced after re-uptake and conjugation of UDCA by the host, and has been shown to reduce inflammation in mice, potentially by preventing the downregulation of nuclear receptors observed under inflammatory conditions^198^. TUDCA has also been observed to affect gut microbiome structure, increasing the relative abundance of Bacillota (formerly Firmicutes) and decreasing Bacteroidota (formerly Bacteroidetes) in mice^199^. However, these findings are in contrast to observations of increased SBA-producing genes in the gut of patients with colorectal cancer^200^. The detrimental impact of SBAs is supported by studies in mice showing that DCA can induce colonic tumour formation^201^.

Currently only a few microbial metabolites and proteins have had their therapeutic potential studied, yet the gut microbiome produces thousands of metabolites and proteins^202^ with more discovered each year^203^. Detailed description of the molecular effector landscape of the gut microbiome will be a major endeavour for coming years. For those molecules that have been studied, standardisation of dosages will facilitate greater understanding of their impact on the ecology of the microbiome as current variation in treatment between studies prevents direct comparison of results.

## Immune modulation by microbiome-based interventions

**Fig. 2:**
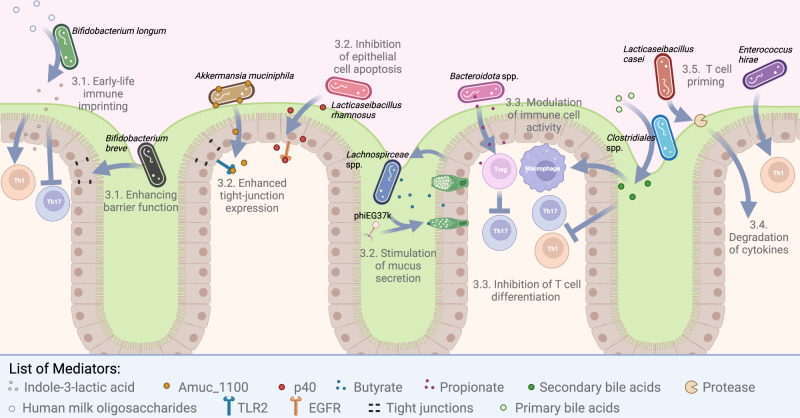
Visualisation of some discussed mechanisms of how microbiome-based interventions can modulate the immune system. Each interaction is numbered based on the sub-section they are detailed in. The key mediators and molecules involved are detailed below the scheme. Species known to conduct these interactions are given as examples in black. The mucus layer is depicted in green. As a schematic, the sizes and location of mediators and microbes have been modified to enhance visualisation.

In this section, we detail some of the known effects of microbiome-based interventions on the immune system. As this is a broad field of research, we have focused this section on interactions for which mechanisms have been studied and those with therapeutic application. Each subsection focuses on a mechanism by which microbial effectors (e.g., FMT) impact immune targets (e.g., cytokine production). Many studies which identify an association between a change in the microbiome, such as one caused by a microbiome-based intervention, and a change in host phenotype fail to provide a mechanisms^204^. Due to the difficulty of conducting mechanistic experiments in humans, we also consider mouse and cell lines experiments to provide greater insights into the pathways and cells involved. However, mechanisms identified in mouse experiments may not transfer to humans, hence their validation is still required in humans^205^. An overview of the discussed mechanisms is shown in Fig. 2.

### Modulation of early-life priming of the immune system

Establishment of the gut microbiome in early life overlaps with the immune priming window. Epidemiological and more recent mechanistic studies indicate that a perturbed perinatal gut microbiome is associated with heightened risk of developing immune-mediated disorders, including atopic dermatitis, asthma, food allergies, chronic intestinal diseases, and autoimmune conditions^206,207^.

Immune priming begins in utero with the mother and can be linked to increased *Bifidobacterium* over the course of pregnancy. Supplementation of germ-free dams with *Bifidobacterium breve* UCC2003 is associated with transcriptional and morphological changes in the placenta and foetal compartments, including alterations of foetal liver immune pathways^208^. Other studies in humans have also indicated that maternal diet, such as fibre, and microbiome shape immune factors in cord blood, being associated with cord blood IgA^209^.

As discussed above, there is a strong evolutionary link between diet, especially human milk, the gut microbiome, and infant health. Components of human milk are known to beneficially drive immune programming and maturation (for a recent review see Singh et al.^210^). Several recent studies have suggested a number of mechanisms by which human milk-*Bifidobacterium* interactions directly influence early life immune responses. A lack of *Bifidobacterium* and associated HMO-metabolism genes is associated with a heightened inflammatory state in infants, including higher levels of neutrophils and basophils (and plasma TNF-α and IL-17A), while infants with higher endogenous *Bifidobacterium* were observed to have had a higher frequency of anti-inflammatory monocytes and regulatory T cells, and elevated levels of circulating IL-10. Direct supplementation with *B. longum* subsp. *infantis* EVC001, which encodes a range of HMO metabolism genes, was associated with modulation of intestinal T cell responses; Th2 and Th1-associated cytokines were reduced to undetectable limits, whilst interferon β (IFNβ) increased in faeces. Moreover, supplemented infants had higher levels of a key metabolite, indole-3-lactic acid (ILA), which exerted direct regulatory effects on Th2 and Th17 cells in vitro, via the induction of regulatory galectin-1, which is known to limit T cell activation (Fig. 2)^211^. Linking to ILA, microbial-derived components and metabolites signal via the aryl hydrocarbon receptor (AhR), a transcriptional factor that regulates the host immune system^212^. Endogenous ligands of AhR are contained in foods such as brassicas and are converted into AhR ligands such as indole and indole derivatives by the gut microbiome. During early life, certain *Bifidobacterium* species in the infant gut can metabolise human milk derived components, i.e., aromatic amino acids (tryptophan, phenylalanine and tyrosine), into their respective aromatic lactic acids (ILA, phenyllactic acid and 4-hydroxyphenyllactic acid) via aromatic lactate dehydrogenases. ILA from *Bifidobacterium* activates AhR in a dose-dependent manner in vitro, and modulated CD4 + T cells and monocytes responses ex vivo^213^. These studies have begun to shed light on how key microbial-derived metabolites modulate developing immune responses in early life, and may provide a key link as to why formula-fed infants (that have reduced levels of *Bifidobacterium*, and HMOs) are, in some cases, at a heightened risk of later life immune-mediated conditions like asthma^214^ given their potentially skewed initial immune priming.

Strengthening of the epithelium barrier during early life is critical to limit unregulated stimulation of the mucosal and systemic immune system that may lead to inflammatory associated conditions^215^. A particular condition of concern during early life is NEC, which impacts between 5–15% of preterm infants. Previous studies in mice have indicated that *Bifidobacterium* plays an oversized role in guiding epithelial barrier development. Specifically, supplementation with *B. breve* UCC203 during the neonatal window was associated with whole scale transcriptional responses in the small intestinal epithelial compartment, with stem cell marker genes being selectively targeted, indicating an increased regenerative potential of the epithelial layer (Fig. 2)^216^. This is important in the context of NEC, as numerous studies have shown that supplementation with *Bifidobacterium* (and *Lactobacillus*) probiotics significantly reduces NEC incidence in preterm infants^217^, including strain EVC001^218^. This links to previous findings in various animal models and ex vivo studies also showing how addition of specific strains of *Bifidobacterium* can reduce intestinal epithelial cell apoptosis and NEC pathology in vivo^219^.

### Modulation of innate immune function

As discussed in the previous section, enhancing barrier function in early life is a key mechanism by which probiotics can help reduce the incidence of NEC, but barrier function is also important in adults. The term ‘leaky gut’ has been used to describe when reduced epithelial barrier function leads to increased occurrence of microbial products within the body^220^. Both probiotics and prebiotics have previously been found to improve barrier function in patients with obesity^221^.

Intestinal permeability is also impacted by SCFAs. In experiments using CaCo-2 cells, *Bifidobacterium bifidum* was shown to reduce the impact of TNF-α induced barrier permeability via production of acetate^222^. In mice, reduced barrier permeability cause by oral acetate supplementation (300 mM in the drinking water beginning two days prior to the experiment and continuing for the duration) reduced susceptibility to DSS colitis^223^. Acetate is not the only SCFA to influence barrier function; butyrate has been shown to improve barrier integrity, but increase permeability at high doses^224^. When administered to mice at 5% of total food weight, given *ad libitum*, butyrate significantly increased colonic expression of occludin, claudin-5, and zonula occludens protein-1 (ZO-1), leading to a significant decrease in intestinal permeability^225^. Cell line experiments have shown that butyrate also upregulates expression of IL-10 receptor α subunit, a molecule shown to be essential for barrier formation^226^.

The probiotic strain LGG secretes two small soluble proteins, named p75 and p40, both of which inhibit cytokine-induced apoptosis in cultured epithelial cells^227^. By activating the epidermal growth factor receptor (EGFR), and its downstream target, Akt, p40 has been identified to prevent apoptosis and maintain barrier integrity in mice (Fig. 2)^228^. This provides a mechanism by which LGG improves intestinal development and tight-junction formation during the first week of life in mice^185^. Production of p40 by LGG can be modulated by the release of extracellular vesicles containing heat-shock protein 90 (HSP90) from host epithelial cells^229^. It has been shown in mice that p40 can be provided as a postbiotic and confer the same benefits, without host factors influencing its expression^184^.

The protein, Amuc_1100, from *A*. *muciniphila* Muc, has also been shown to improve barrier function when applied to CaCo2 cells^230^ via interacting with toll-like receptor 2 (TLR2)^231^. Cell line experiments have also shown that activation of TLR2 can increase the expression of tight junction proteins^232^, which may be the mechanism by which *A*. *muciniphila* improves barrier function (Fig. 2)^233^. In human trials, *A*. *muciniphila* Muc can improve barrier function, as shown by reduced plasma LPS levels, however this effect was only significant when pasteurised cells and not live cells were ingested^163^.

Before microbes can reach the intestinal epithelium, they must pass the mucus layer which forms a physical barrier between the microbes and the host. Decreased secretion of structural components of the mucus layer, such as MUC2, has been associated with the development of inflammatory conditions within humans^234^. The mucin layer can be enhanced in different ways. Butyrate has been shown to increase MUC gene expression when applied directly to goblet cells (Fig. 2)^235^. In mice, increased release of mucin after administration of butyrate causes the mucus-associated microbiome to expand and reduces the impact of infection-induced inflammation^236^. Exposure of mice to bacterial products such as LPS and peptidoglycan enhances mucin production and recovery of the mucin layer after inflammatory intervention^237^, although this is likely to be dependent on the species present. For example, *A*. *muciniphila* is known to degrade mucin^238^, however it also increases the number of goblet cells within the ileum of treated mice^239^. It has been suggested that *A*. *muciniphila* enhances expression of the Wnt/B-catenin pathway, increasing intestinal epithelial cell proliferation, however this has only been shown in chickens^240^. The prebiotic berberine has also been shown to enhance MUC2 expression in mice, leading to increased mucin production and an associated increase in *A*. *muciniphila*^241^. While *A*. *muciniphila* is one of the best studied mucin-associated bacteria, *Bifidobacterium* spp. have been shown to enhance goblet cell activity in mice, reportedly via production and secretion of acetate^242^. Stimulation of mucin production is not limited to bacteria either; the phiEG37k phage has also been shown to stimulate MUC2 expression, reducing the severity of gastrointestinal inflammation in treated mice^243^.

Mononuclear phagocytes, macrophages and dendritic cells (DCs), within the gastrointestinal tract have also been observed to be modulated by microbiota-based interventions. Butyrate can stimulate differentiation of monocytes to macrophages via inhibition of histone deacetylase 3 (HDAC3) and alter activity by increasing antimicrobial peptide production via reducing activity of mTOR kinase^244^. This was confirmed in vivo by giving mice butyrate in their drinking water (150 mM for seven days), after which their colonic macrophages had higher antimicrobial activity than those given a saline solution. In vitro experiments have also shown that conditioned media from probiotic strains, such as LGG, can enhance the rate at which macrophages kill ingested bacteria. This is done via increasing reactive oxygen species (ROS) generation by enhancing the expression of NADPH oxidase^245^. Some LAB strains have been shown to modify the activity of DCs via the induction of tolerogenic properties. When bone marrow-derived dendritic cells, treated with the probiotic strain *L. rhamnosus* LR-32 were transferred into mice, they conferred a protective phenotype on the recipients to 2, 4, 6-trinitrobenzenesulfonic acid (TNBS)-induced colitis due to the higher abundance of tolerogenic DCs^246^. LAB strains found in fermented foods, particularly sauerkraut, produce D-phenyllactic acid, leading to increased serum levels, which is recognised by the HCA_3_ receptor on monocytes, altering their migration^247^.

### Modulation of T cell populations

In addition to modulation of innate immunity, microbiome-based interventions can have profound effects on the adaptive immune system. For example, in patients with end-stage renal disease, daily intake of propionate (1000 mg) was shown to expand the Treg population within the peripheral blood^248^ and has consistently been shown to decrease the levels of C-reactive protein, a marker of inflammation, in peripheral blood by half, although its influence on other inflammation markers (IL-2, IL-17) are inconsistent^249^. Propionate has also been shown to modulate the activity of immune cells, including increased production of IL-10 by Tregs, which leads to inhibition of Th17 cells (Fig. 2). In the context of Multiple sclerosis, patients receiving propionate daily (1000 mg) for 14 days reported alleviation of clinical symptoms and a reduced risk of disease progression if taken continuously over a year^250^.

Modification of bile acids by the gut microbiome produces a wide range of SBAs, many of which impact the immune system and some of which have been applied therapeutically. The SBA lithocholic acid (LCA) is a potent inhibitor of IL-1β production in macrophages via inhibiting activation of the NLRP3 inflammasome. Dominant human gut bacteria, including *Gordonibacter pamelaeae*, *Eggerthella lenta*, and *B. fragilis*, were identified to modify LCA into 3-oxolithocholic acid (3-oxoLCA) and isolithocholic acid (isoLCA)^251^. Both of these metabolites reduced the differentiation of Th17 cells but had no impact on Treg or Th1 cells in human cell lines and mouse models. However, LCA itself has been shown to inhibit differentiation and activation of Th1 cells (Fig. 2)^252^. Taken together, these results suggest that bacterial modification of bile acids, particularly the production of LCA and its derivatives, have a substantial impact on T cell populations and their activity.

Some microbial metabolites have been identified to act as AhR ligands, detailed above, and modulate the T cell population of the gut^253^. Metabolism of tryptophan by the gut microbiome produces indole-3-acetic acid (IAA)^254^, leading to activation of AhR via IL-22 production, improving intestinal recovery in colitis mouse models^255^. IAA-induced IL-22 production is key for colonisation resistance to gastrointestinal fungal infections in mice^256^. While postbiotic treatment with IAA has not been studied in humans, many lactobacilli used as probiotics are IAA producers.

Many other immune cells, other than T cells which have been the focus of this section, are impacted by the gut microbiome. As such, they can be part of the mechanism by which microbiome-based interventions impact the immune system. Recently, probiotic bacterial consortia have been developed to modulate immune populations. GUT-108 is one such consortia which reduces the CD4 + T cell population within the gut, reducing the abundance of multiple inflammatory cytokines^152^. While effective in mice, clinical trials are required to confirm its application in humans and better understand the mechanisms by which it affects the immune system.

### Modulation of cytokine levels

Cytokines are host molecules used for communication between immune cells. Altering the abundance of these molecules can change the migration and activity of specific immune cell types. Gut microbes have evolved multiple methods to modulate the ability of cells to produce and secrete these molecules. Lactocepin is a protease produced by *Lacticaseibacillus casei* which is able to degrade IP-10 (CXCL10), a proinflammatory molecule (Fig. 2)^257^. Based on this, lactocepin-producing probiotic species have been proposed to be of therapeutic use for IBD patients^258^, while no clinical trials studied their application, probiotic use of *L*. *casei* DN-114 001 has proven effective against gastrointestinal inflammatory conditions^259^.

Degradation of inhibitory cytokines, leading to increased activation of the immune system, is another method of interaction. Cgr2 is an enzyme produced by *E. lenta* that can convert digoxin, a cardiac medication, into an inactive molecule^260^. Cgr2 also degrades RORγt inhibitors increasing levels of IL-17a (CTLA-8) and activating Th17 cells within mice, although the specific inhibitor degraded is currently unknown^261^. This proinflammatory process can itself be inhibited via dietary arginine which appears to inactivate Cgr2^262^. Therefore, ingestion of arginine may be a method to modulate Cgr2 activity and reduce gastrointestinal inflammation, as studies in mice have shown^263^. In addition to cytokine degradation, microbes can alter their secretion. For instance, a probiotic consortium altered the ratio of inflammatory to anti-inflammatory cytokines released by Caco2 cells^169^ and when applied to patients with IBS, reduced symptom severity^264^.

Ursodeoxycholic acid (UDCA), commercially called ursodiol, is a SBA which has been used clinically for decades^197^ and inhibits cultured epithelial cells releasing proinflammatory chemokines, therefore reducing the severity of inflammatory responses^265^. However, trials have so far failed to prove their effectiveness to treat conditions such as IBD^266^. As the impact of bile acids is dose-dependent^267^, it may be that an insufficient dose was applied. Additionally, high-dosage treatment with UDCA has also proven detrimental, being linked to increased risk of colorectal neoplasia in patients with ulcerative colitis and primary sclerosing cholangitis^268^.

### Stimulation of immune cells to enhance response to check-point inhibitors

The gut microbiome has been identified as a factor influencing host response to medication, such as check-point inhibitor (CPI) therapy^269^. CPIs are a form of immunotherapy for treatment of cancer which target the immune escape mechanisms used by cancerous cells. There are three main target molecules that are inhibited; CTLA-4, PD-1, and PD-L1. The interaction between the microbiome and CPIs has been shown to be complex and is not limited to a single species, instead showing cohort-specific microbiome signatures^270^.

Based on this, FMT has been used to modulate the microbiome of non-responders and improve CPI therapy. This was successful in 30% of patients (*n* = 10) treated with anti-PD-1 CPI therapy^271^ and further improved to 40% when using faecal material from responders^272^. While the exact mechanism by which FMT improves anti-PD-1 response is undefined, the microbiome-dependent nature of CTLA-4 CPI therapy has been better characterised. *B. fragilis* and *Bacteroides thetaiotaomicron* were identified to be key for T cell priming, without which CTLA-4 blockade is ineffective (Fig. 2). Specifically, the zwitterionic polysaccharides found in the capsule of these species stimulates the immune response. Responders have also been confirmed to have significantly greater abundance of these taxa within their gut microbiome^273^. Increasing the abundance of these species via application of microbiome-based interventions may improve response to CTLA-4 CPI, however, such methods should be targeted as other members of this genus (*Bacteroides*) are not beneficial to the host^274^. *Bacteroides intestinalis* has been linked to toxicity during combined CPI therapy^275^. This suggests the interactions between this genus of bacteria and the immune system are complex and require further study to ensure the best outcome for the patient.

Other members of the gut microbiome associated with clinical response to PD-1, and PD-L1 treatment are *A. muciniphila* and *Enterococcus hirae*. Germ-free mice gavaged with these species were observed to become responsive to PD-1 treatment. This may be in part due to stimulating the secretion of IL-12 by DCs, Th1 cell reactivity to *A*. *muciniphila*, and Tc cell reactivity to *A. muciniphila* and *E. hirae*^276^, however the exact mechanism remains unclear.

## Conclusions

While microbiome-based interventions influence the host immune system using multiple ways, our understanding is still in its infancy and further work is needed to understand these mechanisms and utilise this information to design better intervention strategies. However, implementation of this information has already led to great advancements over a relatively short period of time and future advancements are likely to occur at a fast rate. In Box 2 we detail a few of the most exciting developments in recent and coming years. Additionally, while this review has focused on the bacterial and viral fractions of the microbiome, the fungal residents of the gut have also been identified as being important to host health and their potential use should be studied further^277^.

Box 2 Future development of microbiome-based interventions

- Genetic modification of commensal strains to produce additional molecules such as IL-10^301^, butyrate^302^, as well as enzymes such as bile salt hydrolase, allowing for the modification of bile acid metabolism, may enhance the strains beneficial properties as a probiotic. However, such modified strains have not yet been tested in humans^161^. Further analysis of factors influencing strain engraftment or sensitivity to drugs may also improve probiotic design.
- Advances in bioinformatic approaches combined with cultivation efforts may enable the selection of microbial consortia usable as personalised probiotic mixtures, designed to treat the condition of interest^303^.
- Using our knowledge of each species metabolic requirements, generation of open niches has been proposed as a method to achieve long-term engraftment. By supplementing nutrients that few species can utilise, engraftment of specific strains can be achieved^304^.
- Modification of how probiotics are prepared and administered has also advanced in recent years. One such advancement is nanocoating of probiotics in a mixture of tannic acid and ferric ions confers some resistance to antibiotics. In mice, this allows the probiotic to colonise during antibiotic treatment, reducing adverse effects of antibiotic treatment such as weight loss and diarrhoea^305^.
- The evolutionary trajectory of gut microbes can also be directed via modification of the hosts immune response, such as stimulation of specific IgA responses^306^. While this has only been applied to pathogens to reduce colonisation, a similar approach could be applied to enhance colonisation of beneficial commensals.
- By designing food, not only for the host, but for the microbiome, host-microbe interactions can be utilised to enhance host health^290^. Microbiota-directed complementary food (MDCFs) applied this principle and have been shown to modify the microbiome and lead to improve the growth of undernourished children^291^.
